# Deep progressive learning achieves whole-body low-dose ^18^F-FDG PET imaging

**DOI:** 10.1186/s40658-022-00508-5

**Published:** 2022-11-22

**Authors:** Taisong Wang, Wenli Qiao, Ying Wang, Jingyi Wang, Yang Lv, Yun Dong, Zheng Qian, Yan Xing, Jinhua Zhao

**Affiliations:** 1 Department of Nuclear Medicine, Shanghai General Hospital, Shanghai Jiaotong University School of Medicine, No. 100 Haining Road, Shanghai, 200080 People’s Republic of China; 2 United Imaging Healthcare, Shanghai, People’s Republic of China

**Keywords:** PET, Deep progressive learning, Low dose, Image quality

## Abstract

**Objectives:**

To validate a total-body PET-guided deep progressive learning reconstruction method (DPR) for low-dose ^18^F-FDG PET imaging.

**Methods:**

List-mode data from the retrospective study (*n* = 26) were rebinned into short-duration scans and reconstructed with DPR. The standard uptake value (SUV) and tumor-to-liver ratio (TLR) in lesions and coefficient of variation (COV) in the liver in the DPR images were compared to the reference (OSEM images with full-duration data). In the prospective study, another 41 patients were injected with 1/3 of the activity based on the retrospective results. The DPR images (DPR_1/3(p)) were generated and compared with the reference (OSEM images with extended acquisition time). The SUV and COV were evaluated in three selected organs: liver, blood pool and muscle. Quantitative analyses were performed with lesion SUV and TLR, furthermore on small lesions (≤ 10 mm in diameter). Additionally, a 5-point Likert scale visual analysis was performed on the following perspectives: contrast, noise and diagnostic confidence.

**Results:**

In the retrospective study, the DPR with one-third duration can maintain the image quality as the reference. In the prospective study, good agreement among the SUVs was observed in all selected organs. The quantitative results showed that there was no significant difference in COV between the DPR_1/3(p) group and the reference, while the visual analysis showed no significant differences in image contrast, noise and diagnostic confidence. The lesion SUVs and TLRs in the DPR_1/3(p) group were significantly enhanced compared with the reference, even for small lesions.

**Conclusions:**

The proposed DPR method can reduce the administered activity of ^18^F-FDG by up to 2/3 in a real-world deployment while maintaining image quality.

**Supplementary Information:**

The online version contains supplementary material available at 10.1186/s40658-022-00508-5.

## Introduction

Positron emission tomography (PET) is a noninvasive molecular imaging modality widely used in oncology, neurology, cardiology, and other fields [1–8]. Good image quality and accurate quantification are vital in PET imaging for clinical diagnosis, prognosis, staging/restaging, and treatment monitoring. Nowadays, the ordered subset expectation maximization (OSEM) algorithm is the most popular PET imaging method used in clinical routines. A major drawback of the OSEM algorithm is that the image noise increases fast as the iteration number grows, so the iterative process has to be stopped early, and therefore, an additional post-processing filter is used to smooth the image. Advanced reconstruction algorithms such as BSREM [9] and TVREM [10] incorporate a penalty term in the formula which suppresses the image background noise during the iterative reconstruction process and thus no post-processing filter is needed. During the past few years, deep learning approaches have been proven to achieve superior performance in denoising PET images [11–16]. It has shown the potential of low-dose to full-dose conversion in various studies. Sanaatet al. predicted regular full-dose PET images from simulated 1/8th low-dose PET images using CycleGAN model [11]. Schaefferkoetter et al. evaluated the denoising performance of a 3D CNN model with different count levels from 1 to 20 million [12]. Wang et al. developed a CNN model which combines simulated 1/16th low-dose PET scans and simultaneously MRI scans to produce a standard-dose PET scan [13]. Mehranian et al. trained the deep learning enhancement model with partial-duration OSEM images into target full-duration BSREM images and finally allowed a reduction in scan time by up to 50% [14]. The main concerns of previous studies are as follows: (i) The training target images were acquired from conventional PET scanners with a long acquisition time, which may provide PET images with good but sub-optimal image quality because the sensitivity of the conventional PET scanners is much lower than that of the total-body PET scanner; (ii) all studies use deep learning as an image post-processing technique; however, recent studies have shown that incorporating the neural network model into the iterative process may achieve better results [15], 16; and (iii) though all the studies aim to realize low-dose PET body imaging, the evaluations were conducted using simulated data, not real-world low-dose data.

We have developed a deep progressive learning reconstruction method (DPR) and evaluated it with extensive phantom and patient studies [16]. The training data used in the network come from the total-body PET (uEXPLORER, United Imaging Healthcare, China) images with an acquisition time of 15 min, which can generate excellent image quality and can easily overcome the above-mentioned challenges. However, uEXPLORER is too expensive and not every PET center can afford it. If the training data acquired from uEXPLORER can be used to improve the image quality of conventional PET scanners, the entire molecular imaging community can benefit from it. Thus, we designed this study and validated it using uMI 780, a digital PET/CT scanner with a standard axial field-of-view (FOV) of 30 cm. We first investigated the performance of the DPR algorithm in shortening the acquisition time with the retrospective data. The reduced acquisition is to simulate the reduced injected activity in PET imaging and provide evidence for the subsequent study with real-world low-dose injection. Thus, the patients were prospectively enrolled with an injection of reduced activity based on the above results. The image quality of these patients was comprehensively evaluated regarding quantification accuracy, lesion contrast as well as visual assessment.

This study aimed to investigate the image quality of ^18^F-FDG PET images reconstructed by the DPR algorithm in patients with both a simulated and real-world low-injected activity and compare it to that reconstructed by the standard OSEM algorithm.

## Methods

**Fig. 1:**
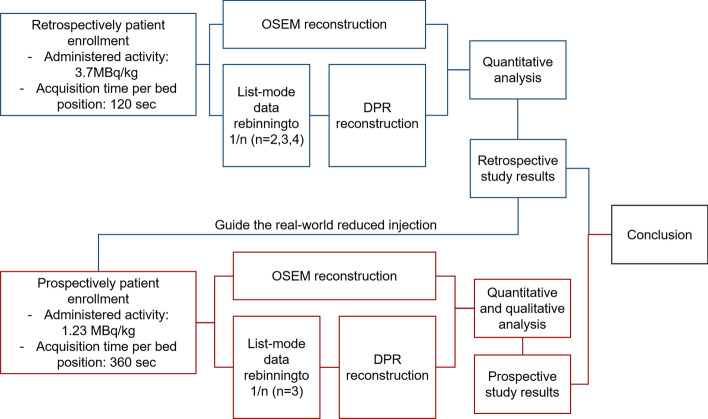
Flow diagram of the reconstruction process and analysis. OSEM, ordered subset expectation maximization algorithm. DPR, deep progressive learning algorithm

The DPR algorithm employs two networks, i.e., a denoising network (CNN-DE) which can remove the noise from the input image and an enhancement network (CNN-EH) which maps from a low convergent image to a high convergent image. Both CNN-DE and CNN-EH were trained based on the designed feedback network. One hundred patient data acquired from uEXPLORER were used for network training. For CNN-DE, PET images with 10% uniformly down-sampled counts were used as training input, and PET images with full counts were used as training targets. For CNN-EH, PET images with insufficient iterations were used as training input, and PET images with sufficient iterations were used as training targets. The details of algorithm design, network training and testing are described in the Additional file 1. The algorithm was evaluated in two steps, and a flow diagram of the study is shown in Fig. 1. A current guideline for PET oncological study [1] recommends the specified product of acquisition time per bed position and the administered activity for a given patient. Therefore, one may decide to apply a higher activity and reduce the acquisition duration, or to use a reduced activity and accordingly increase the acquisition duration. The latter one is preferable to keep in line with the ALARA principle. In our clinical routines, the acquisition time for each bed position is fixed as 2 min with an administered activity linear to the patient weight (3.7 MBq/kg) to simplify the acquisition procedure. Thus, the performance of the proposed DPR algorithm was first evaluated using the retrospective data where the above dose regimen was applied. The acquisition time per bed position was reduced to 1/n (*n* = 2, 3, 4) to simulate the low-dose scenario. Quantitative analysis was performed to compare the quantification accuracy and image quality between the full-dose data reconstructed with OSEM and low-dose data reconstructed with DPR and evaluate the ability of the DPR in reducing the dose. Based on the results from the retrospective study, the patient was prospectively enrolled and injected with reduced activity in the subsequent prospective study. Both the qualitative and quantitative analyses were performed to compare the full-acquisition data reconstructed with OSEM and reduced-acquisition data reconstructed with DPR.

### Patients

The study included a retrospective and a prospective patient cohort. Twenty-six and 41 oncological patients (female/male: 26/41, age 15–87 years) referred to the Shanghai General Hospital from November 2020 to September 2021 for clinical ^18^F-FDG PET/CT examinations were enrolled, respectively. Their demographic and clinical information is listed in Table 1. All patients had fasted for at least 6 h, and a blood glucose level was confirmed to be ≤ 10 mmol/mL before the ^18^F-FDG injection. A weight-based ^18^F-FDG (full and one-third) dose was administered to the patient of the two cohorts using a fully automated PET infusion system (MEDRAD, Bayer Medical Care Inc. Pennsylvania, USA) that allows an accurate dose administration. During an uptake period of about 60 min, the patients were hydrated orally with 0.5–1.0 L of water. This study was approved by the Institutional Review Board of Shanghai General Hospital, and the written informed consent was waived in the retrospective part and obtained from the patients in the prospective part.

**Table 1:** Patient demographic characteristics

Parameter	Retrospective study	Prospective study	*p* value
No. of patients	26	41	N.A.
Age (years)	63.7 ± 16.4 [24, 87]^a^	52.9 ± 12.2 [15, 69]^a^	0.003
Gender			
Female	8	18	0.315
Male	18	23	
Weight (kg)	65.2 ± 12.2 [51.2, 110]^a^	61.9 ± 12.6 [44, 101.4]^a^	0.233
Height (cm)	166.2 ± 8.6 [150, 184]^a^	163.4 ± 9.6 [146, 183]^a^	0.289
BMI	23.5 ± 3.1 [17.3, 32.5]^a^	23.0 ± 3.4 [16.3, 31.7]^a^	0.570
Glucose level	5.38 ± 0.88 [4.3, 7.9]^a^	5.34 ± 0.84 [3.8, 8.1]^a^	0.846
Injected activity (MBq)	258 ± 46 [200, 407]^a^	81 ± 15 [51, 115]^a^	< 0.001
Injected activity/weight(MBq/kg)	3.97 ± 0.28[3.61, 4.75]^a^	1.32 ± 0.18[1.10, 2.11]^a^	< 0.001
Uptake time (min)	70 ± 14 [49, 97]^a^	68 ± 16 [39, 105]^a^	0.458
Primary cancer type			N.A.
Leukemia	1^b^	3^b^	
Bladder cancer	1^b^	0	
Multiple myeloma	1^b^	0	
Lung cancer	6^b^	9^b^	
Liver cancer	0	1^b^	
Laryngeal carcinoma	1^b^	0	
Bone tumor	1^b^	0	
Colorectal cancer	3^b^	2^b^	
Lymphoma	5^b^	8^b^	
Ovarian cancer	1^b^	0	
Breast cancer	1^b^	2^b^	
Renal cancer	1^b^	1^b^	
Esophageal cancer	2^b^	2^b^	
Gastric cancer	1^b^	0	
Pancreatic cancer	1^b^	1^b^	
Pelvic carcinoma	0	1^b^	
Tongue cancer	0	2^b^	
Uterine cancer	0	5^b^	
Aplastic anemia	0	1^b^	
Popliteal fossa tumor	0	1^b^	
Tonsil tumor	0	1^b^	
Nasopharyngeal cancer	0	3^b^	

N.A., not applicable; BMI, body mass index

^a^Data are presented as the mean ± standard deviation [minimum, maximum]

^b^Number of patients

### PET/CT acquisition and reconstruction

All patients were scanned with a digital PET/CT scanner (uMI 780, United Imaging Healthcare). The system details and performance characteristics of uMI 780 and uEXPLORER are summarized in Table 2. These two systems have a similar spatial resolution, energy resolution and TOF resolution. The only major difference is the axial FOV, which leads to the sensitivity difference. Patients were firstly scanned with CT with a fixed tube voltage of 120 kV and an auto-mAs technique for dose modulation, providing anatomical information and attenuation correction to PET images. Subsequently, patients were scanned with PET in step-and-shoot mode. PET data were acquired in list mode for 2 min per bed position in the retrospective study. In the prospective study, the patients were injected with one-third of the activity and the acquisition duration was accordingly increased to 6 min to achieve the same product of the administered activity and the acquisition duration as that in the retrospective study. The acquired data were reconstructed with both OSEM and DPR algorithms (hereinafter referred to as OSEM_full(r) and DPR_full(r) in the retrospective study, and OSEM_full(p) in the prospective study). The OSEM algorithm was applied with 2 iterations, 20 subsets, a Gaussian filter with full width at half maximum of 3 mm, 192 × 192 matrix, 600 FOV, 2.68 mm slice thickness, as well as TOF and resolution modeling. The standard corrections (scatter, random, dead time, decay, attenuation, and normalization) were included in the reconstruction. The DPR algorithm was applied with the same FOV, matrix, and slice thickness as in the OSEM. No other post-processing method was included in the reconstruction.

**Table 2:** PET scanner characteristics of uMI 780 and uEXPLORER

	uMI 780	uEXPLORER
Crystal material	LYSO	LYSO
Crystal size	2.76 × 2.59 × 18 mm^3^	2.76 × 2.76 × 18 mm^3^
Number of detector rings	112	672
Detector	SiPM	SiPM
Axial FOV	300 mm	1940 mm
Sensitivity	16 kcps/MBq	176 kcps/MBq
TOF resolution	450 ps	430 ps
Spatial resolution	2.9 mm@center	2.9 mm@center
Scatter fraction	38%	38%

Data are from the datasheets provided by the manufacturer

In the retrospective study, we rebinned the list-mode PET data to 1, 2/3, and 1/2 min per bed position to simulate the 1/n (*n* = 2, 3, 4) of the administered activity. The DPR algorithm was also applied to the rebinned PET data (hereinafter referred to as DPR_1/2(r), DPR_1/3(r) and DPR_1/4(r)). In the prospective study, PET images were reconstructed using the first 2 min data with the DPR algorithm (hereinafter referred to as DPR_1/3(p)). In summary, OSEM_full(r) was compared to DPR_full(r), DPR_1/2(r), DPR_1/3(r), and DPR_1/4(r) in the retrospective study, while OSEM_full(p) was compared with DPR_1/3(p) in the prospective study.

### Image analysis in the retrospective study

In the retrospective study of this work, the image quality was quantitatively assessed on an advanced workstation (uWS-MI, United Imaging Healthcare). For each patient, a volume of interest (VOI) with a diameter of 30 ± 3 mm was manually drawn at the same position and the slice on a homogeneous area of the right liver lobe. The SUVmean and standard deviation (SD) within the VOI were recorded. The liver COV, as a measure of background noise, was obtained by dividing the SD by the SUVmean. Regarding the lesions, SUVmax of the identified FDG-avid lesions was measured by placing a VOI to encompass the whole lesion. Thus, tumor-to-liver ratio (TLR), as a measure of image contrast, was obtained by dividing the lesion SUVmax by the liver SUVmean.

### Image analysis in the prospective study

In the prospective study, the same nuclear medicine physician analyzed the images on the same workstation as in the retrospective study. Similarly, a VOI was manually drawn at the right liver lobe, aorta, and gluteus maximus. The SUVmean and standard deviation (SD)of the VOI for each series were recorded. The liver and the muscle COV were obtained by dividing the SD by the SUVmean. The value of SUVmax and TLR for each identified lesion was obtained using the same method as the above. In addition, the diameter of the lesion was measured on the CT images.

Subsequently, the qualitative image quality of unlabeled images was assessed by two nuclear medicine physicians (XY, 17 years of experience, and WTS, 18 years of experience) in a randomized order. The patient’s clinical information, as well as the acquisition duration and reconstruction algorithm, were blinded to the reader. The physicians viewed both the maximum intensity projection (MIP) and transverse PET images and judged the image quality using a 5-point Likert scale in the following three perspectives: image contrast, image noise and diagnostic confidence (with 1 = worst and 5 = best). A score of 3 was given to images that were acceptable for clinical diagnosis.

### Statistical analysis

Continuous parameters are presented as the mean ± SD and range. Fisher’s exact test was performed to investigate the distribution of gender in the two cohorts. An independent *t* test was used to test the other demographic parameters of the enrolled patients from the two cohorts. Fully acquired data were reconstructed with OSEM to generate images as a reference. Bland–Altman plot analyses were performed to assess the agreement of the SUVs between the reference and DPR images. All the quantitative parameters were tested for normality using the Kolmogorov–Smirnov test and the two-tailed paired samples *t* test was subsequently performed. Inter-rater reliability was evaluated using Cohen's weighted kappa (linear) coefficient. The scores of the qualitative image quality were subsequently compared using the Wilcoxon signed-rank test. Statistical significance was considered for a p value less than 0.05, and all statistical tests were performed using SPSS Statistics, version 25 (IBM, Armonk, NY, USA) and R package.

## Results

### Retrospective study

**Fig. 2:**
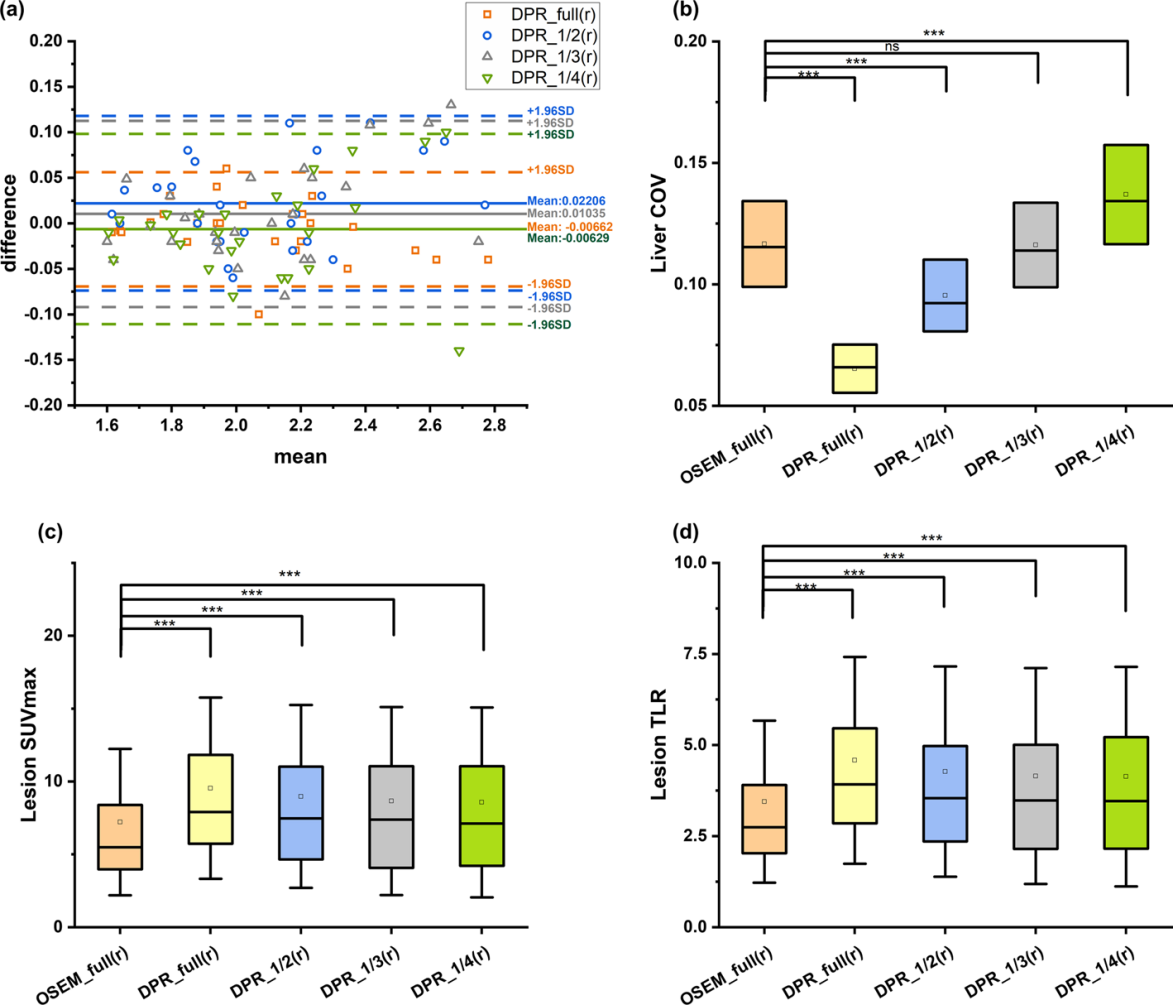
Quantitative results of the retrospective study. **a** is Bland–Altman plots of liver SUVmean between the DPR group and the reference group in the retrospective study. All the DPR groups showed a good agreement on the quantification accuracy of the liver SUVmean with the reference. **b**–**d** are the comparison of the liver COV, lesion SUVmax and TLR between DPR groups and the reference in the retrospective study. The liver COV showed no significant difference between the DPR_1/3(r) group and the reference. All the DPR groups show a significantly elevated SUVmax and TLR compared to the reference group, indicating improved image contrast. ***p < 0.001; ns, no significant difference. Whisker indicates a standard deviation. COV, coefficient of variance. SUV, standardized uptake value. TLR, tumor-to-liver ratio

The liver SUVmean agreed well as shown in Bland–Altman plots (Fig. 2). No significant difference was found between the DPR groups and the reference (all *p* > 0.05), indicating a good quantification accuracy. The liver COV in the DPR_1/3(r) group showed no significant difference with that in the reference (*p* = 0.955), indicating a comparable image quality (Fig. 2). The image quality of the DPR_1/2(r) and DPR_full(r) groups was significantly improved with a reduced COV value (both *p* < 0.001). Both the lesion SUVmax and TLR in all the DPR groups showed a significant enhancement compared to those in the reference (all *p* < 0.001), indicating an improvement in lesion conspicuity. Based on the above results, we concluded that the DPR algorithm can reduce the acquisition time to 1/3. Thus, in the subsequent prospective study, the image quality of the patients injected with 1/3 of ^18^F-FDG was analyzed.

### Prospective study

**Fig. 3:**
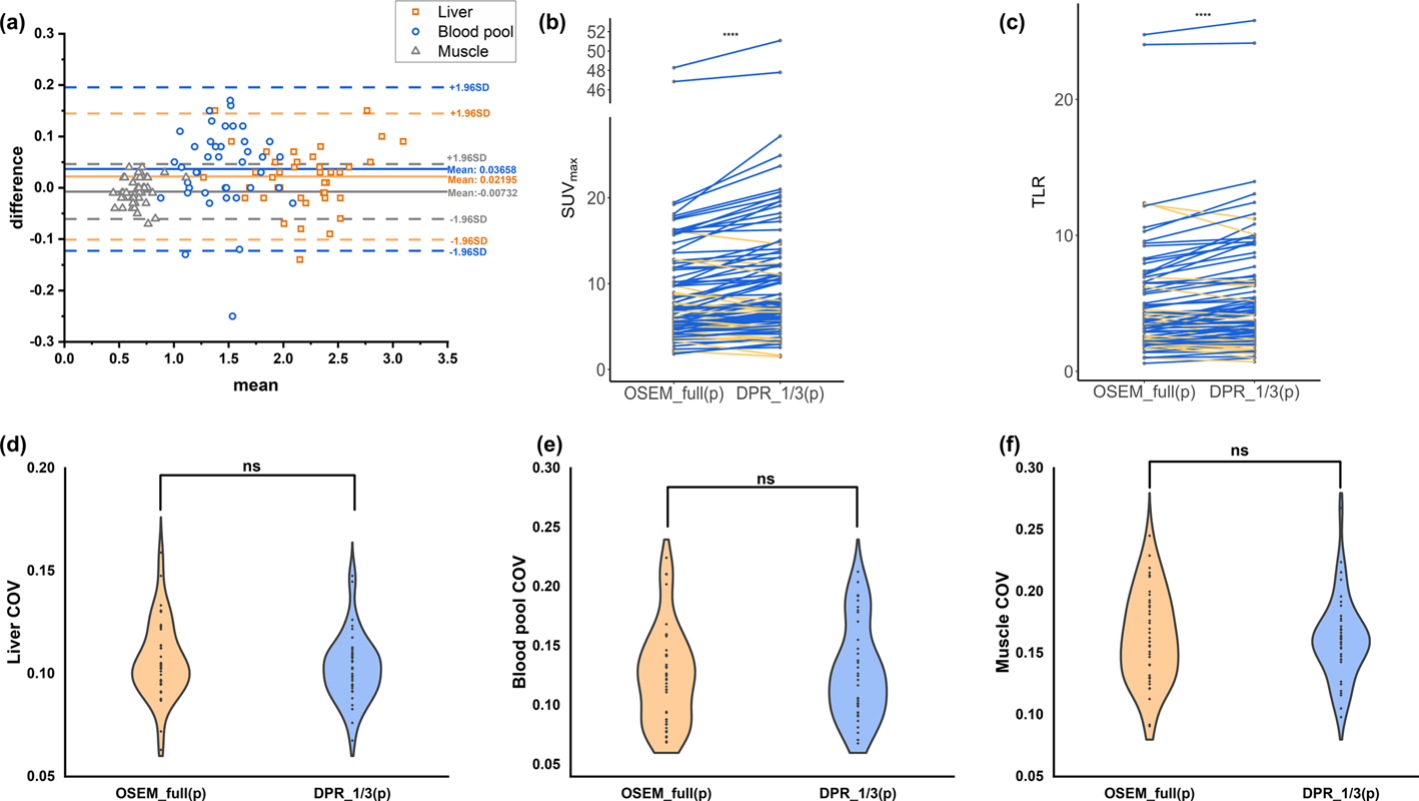
Quantitative results of the prospective study. **a** is the Bland–Altman plots of the liver, the blood pool and the muscle SUVmean between DPR_1/3(p) images and the reference. **b**, **c** are the comparisons of the lesion SUVmax and TLR between DPR groups and the reference (blue line: an increase; orange line: a decrease). The SUVmax and TLR of all the DPR groups were significantly improved compared to the reference group. **d**–**f** are the comparisons of the liver, the blood pool and the muscle COV between DPR groups and the reference. The liver, the blood pool and the muscle COV showed no significant difference between the DPR_1/3 group and the reference. ****p < 0.0001; ns, no significant difference. SUV, standardized uptake value. TLR, tumor-to-liver ratio. COV, coefficient of variance

In the prospective study, we analyzed the uptake of background tissues, including the liver, blood pool and muscle, and found that the values of SUVmean agreed well between groups as shown in the Bland–Altman plots (Fig. 3). Subsequently, the COVs of the background tissues were compared (Fig. 3). There was no significant difference of the COVs between the DPR_1/3(p) group and the reference (*p* = 0.055, 0.526 and 0.604 for the liver, blood pool and muscle, respectively), showing a comparable quality with the reference.

**Fig. 4:**
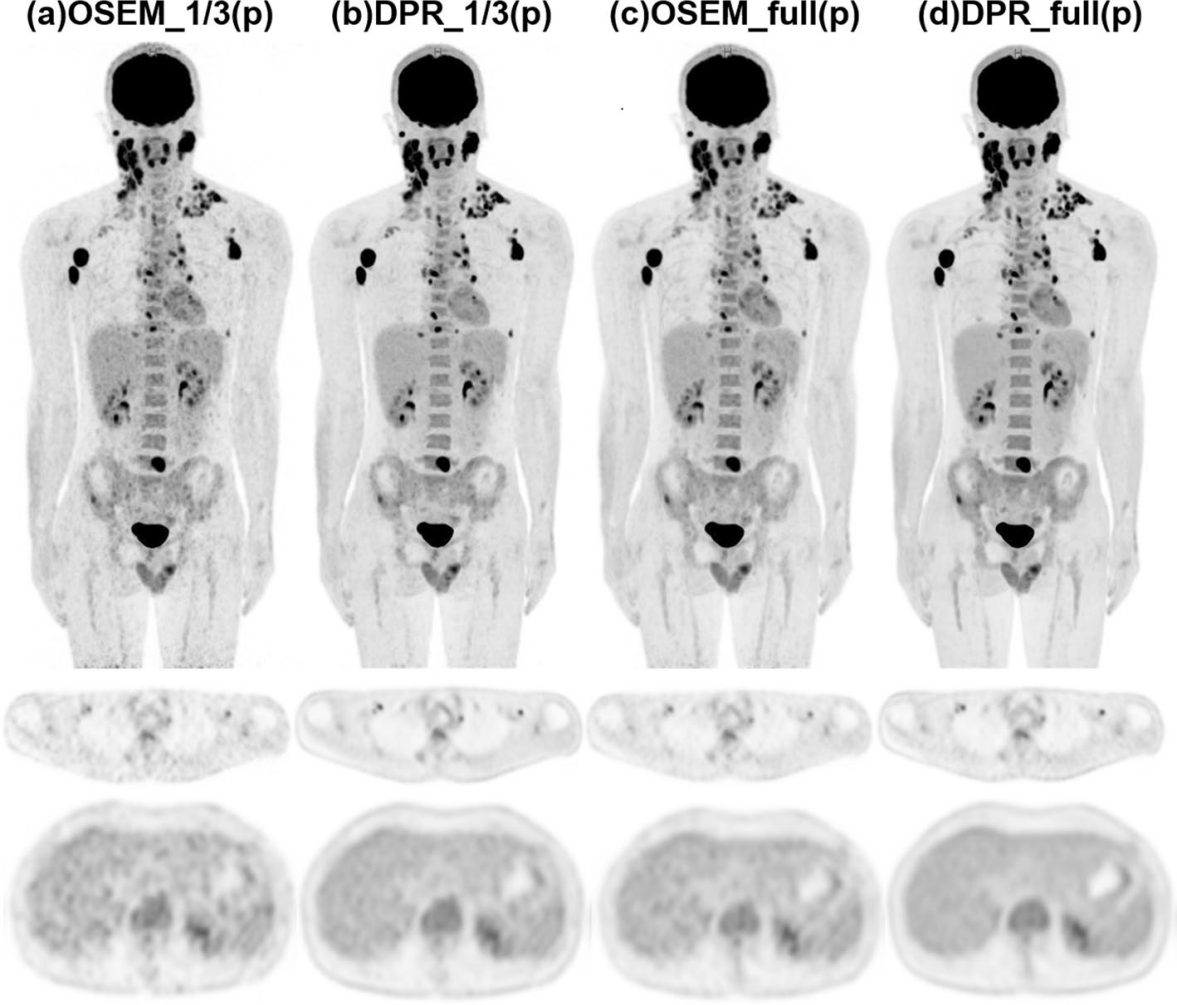
PET images of a 31-year-old male patient diagnosed with Hodgkin's lymphoma in the prospective study. After injection of 85 MBq ^18^F-FDG, the patient underwent a PET scan with 6 min per bed position 77 min post-injection. To comprehensively compare the clinical images reconstructed by the two algorithms, we further reconstructed the data with 1/3 and full of the acquisition duration for OSEM and DPR algorithms. Both the MIP (upper row) and transverse images (middle row) demonstrated improved lesion detectability of the DPR images compared to the OSEM images with the same acquisition duration (**a**–**d**). The transverse image in the liver plane showed reduced image noise when comparing subfigures **a**–**d**, while the image noise in the subfigures b and c was at a comparable level

A total of 98 lesions were identified in the reference images and included in the quantitative analysis. The SUVmax and TLR of the lesions in all the DPR groups were significantly larger than those in the reference group (both *p* < 0.001, as shown in Fig. 3). The enhancement of the lesion uptake in the DPR images can be observed in the MIP and transverse images. Figure 4 illustrates PET images of a 31-year-old man with Hodgkin’s lymphoma. Both the MIP and transverse images of the DPR images showed improved lesion detectability. Meanwhile, the DPR images demonstrated a non-inferior performance in the noise level compared to the reference.

A further study was performed on the small lesions with a diameter of less than 10 mm (*n* = 27). In the DPR images, a significantly higher uptake was also found than that in the OSEM images (*p* < 0.001). Likewise, the TLR in the DPR groups showed significant improvement compared with that in the OSEM group (both *p* < 0.001).

**Fig. 5:**
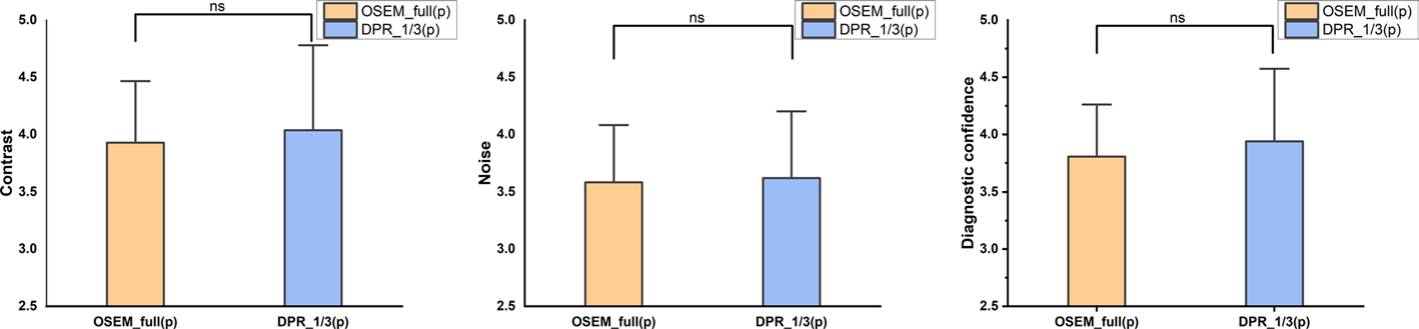
Visual assessment of the image contrast, noise and diagnostic confidence between DPR_1/3(p) images and the reference. There was no significant difference found in all three perspectives. ns, no significant difference

In the visual analysis, the weighted kappa coefficient was 0.612, indicating a substantial agreement of the subjective score between the two readers. There were no significant differences between the DPR_1/3(p) group and the reference regarding the contrast, noise and diagnostic confidence (*p* = 0.284, 0.655 and 0.137, respectively), as shown in Fig. 5.

## Discussion

In this study, we investigated the image quality of a deep progressive learning algorithm with both simulated and real-world reduced administered activity of clinical data. As known, PET is associated with the detection of annihilation photons that are produced back-to-back after positron emission from a radioactive tracer. Hence, radiation exposure is inevitable in PET imaging, and the injected dose should be reduced while maintaining adequate image quality to provide sufficient clinical information. Particularly for longitudinal studies when multiple PET/CT scans are performed, it is desirable to adapt the injected dose to the lowest level to reduce the accumulated dose. In addition, concerns about radiation exposure are of great interest in pediatric populations because the risk of radiation-induced carcinogenesis is higher in children, so they are prone to secondary tumors during their lifetime [17–19]. Thus, reducing the administered activity in PET imaging is highly desirable in clinical routines.

The advent of the total-body or long axial FOV PET scanners has made a breakthrough in the system sensitivity, which has been proven its ability in reducing the administered activity [20–24]. However, up to now, there are only a limited number of total-body PET/CT scanners available worldwide. To better exploit the potential of this powerful tool, a deep learning-based method utilizing the high-quality total-body PET images is expected. Thus, it can be implemented in other PET sites for more urging low-dose PET imaging. The DPR algorithm in this study is the first attempt to utilize the total-body PET images as the target images during the network training. It can be regarded as a bridge to link the conventional PET scanner and the total-body PET scanner. In this work, both the simulated and the real-world low-dose DPR images were evaluated and compared with the standard OSEM images and showed that the image quality can be maintained even if the injected dose was reduced to 1/3.

During the network training of deep learning-based PET denoising techniques, high-quality images reconstructed from a high injected dose or a long acquisition time are required to be used as the training labels. However, high injected dose in PET imaging is related to the potential safety concerns and is not employed in clinical practice. On the other hand, a longer acquisition time may adversely degrade the image quality due to the patient’s motion during the acquisition. An alternative solution is to adapt the regularized images with less noise and better contrast, such as block sequential regularized expectation maximization (BSREM) [14, 25, 26]. Moreover, the employment of the total-body PET images can make an unprecedented breakthrough in the image quality of the training dataset. The proposed DPR method utilized two learning steps to transfer the low-quality images to high-quality images and is feasible for both reducing noise and improving image contrast. A shortcoming of the CNN-based methods is that the performance is usually degraded on small lesions since they are overwhelmed by the image noise in low SNR images. The proposed DPR method can tackle this problem by incorporating the networks into the iteration process [16]. In a previous study on the same network, even the smallest hot sphere with a diameter of 10 mm in a NEMA IEC body phantom still showed at least twofold contrast-to-noise (CNR) gain. Consistent results were found with the clinical data in this study. The DPR algorithm on the small lesions still showed good performance regarding both the quantitative SUV measurements and TLR values.

However, increased lesion SUVmax may have its side effect, that is, the quantitation inconsistency between DPR and OSEM. Many clinical criteria, such as Deauville score on lymphoma, are based on SUV, so increased SUV may lead to different diagnostic results and therefore medical treatment [27]. New PET scanners have better TOF resolution, smaller detector size and higher system sensitivity. The introduction of new reconstruction methods makes the contrast recovery of small lesions higher. As we can see, all advances in hardware and algorithms may have an impact on the detectability of small lesions and therefore change the SUVs. If the quantitative interpretation criteria are not updated with the evolution of PET technologies, it will be a dilemma to adopt new technologies in clinical practice [28, 29]. According to EANM’s recommendations, at this stage, at least two images should be generated for a routine PET examination, i.e., one maximizes the algorithm performance for optimal visual interpretation and the other one follows the EARL specifications for consistent quantitative measurement [1].

The present study has several limitations. First, this work was a single-center study with a limited number of enrolled patients. A large-scale multicenter study is expected to be performed, especially on the quantification accuracy of SUVs which is essential in the multicenter or cross-machine PET study. In addition, the difference in physiological uptake was accidentally observed between the images reconstructed by the first two-minute data and the last two-minute data in the prospective study. In this study, we rebinned the first two-minute data to reconstruct DPR images for analysis. Furthermore, the supervised network in the work was trained with ^18^F-FDG PET data and this study only enrolled oncological patients who underwent ^18^F-FDG PET examinations. Currently, PET imaging combined with other tracers is widely used and studied. In future work, the performance of the DPR algorithm can be trained and evaluated with other non-^18^F-FDG tracers, such as ^68^Ga-PSMA.

In conclusion, in this work, a total-body PET-guided deep progressive learning method was proposed for reducing the noise and improving the contrast of ^18^F-FDG low-dose PET images. Both simulated and real-world low-dose studies demonstrated that PET imaging with the DPR algorithm can reduce the administered activity to 1/3 of the standard without compromising the image quality and small lesion detectability. This study has shown the potential of the proposed DPR algorithm in low-dose PET imaging for conventional digital PET/CT scanners in clinical routines.

## Supplementary Information

**Additional file 1.** The details of algorithm design, network training and testing.

## Data Availability

Data are available upon request to the corresponding author.

## Abbreviations

CNN: Convolutional neural network
COV: Coefficient of variation
DPR: Deep progressive learning reconstruction method
OSEM: Ordered subset expectation maximization
PET: Positron emission tomography
SD: Standard deviation
SUV: Standard uptake value
TB-PET: Total-body positron emission tomography
TLR: Tumor-to-liver ratio
VOI: Volume of interest
