# Supplementary material for: Deep progressive learning achieves whole-body low-dose 18F-FDG PET imaging
Source: EJNMMI Phys. 2022 Nov 22;9:82. doi: 10.1186/s40658-022-00508-5 (PMC9681960; doi:10.1186/s40658-022-00508-5)
Supplement: Supplementary file 1 — Additional file 1. The details of algorithm design, network training and testing. [file 40658_2022_508_MOESM1_ESM.docx]

### Additional File: The details of algorithm design, network training and testing

### DPR algorithm

The problem of PET image reconstruction is to recover an image $x\in R^{N}$ from the noisy linear measurement $y\in R^{M}$ of the form

$y=Ax+r+e$ (1)

where$A\in R^{M\times N}$is the system matrix, $r$ is the random and the scatter estimate, and $e$ is the additive noise. In the framework of the unrolled method for deep learning based iterative image reconstruction algorithms [19], the maximum likelihood estimate of the unknown image $x$ can be calculated as

$\hat{x}=\mathop{\text{arg}\max}\limits_{x} \{L(y|x)-\frac{\lambda}{2}\left\|x-z\right\|^{2}\}$ (2)

where $L(y|x)$ is the log-likelihood function, and $z=f(x ;\theta)$ is a convolutional neural network (CNN) representation of an image $z$ with input image $x$ and parameters $\theta$. The DPR algorithm suggests that the network $f$ could be decomposed into many sub-networks to make the network training easier. Our current implementation employs two sub-networks, that is,

$f\text{ = }f_{DE}\circ f_{EH}$, (3)

where $f_{DE}\text{：}x+\nu\to x$ represents the denoising network (CNN-DE) which can remove the noise $\nu$ from the input image, and $f_{EH}\text{：}x^{(k)}\to x^{(k+q)}$ represents the enhancement network (CNN-EH) which maps from a low convergent image $x^{(k)}$to a high convergent image $x^{(k+q)}$. Both CNN-DE and CNN-EH are trained based on the designed feedback network (FB-Net), as shown in Fig. 1. The result of the third branch is considered to be the output of the network, which is a denoised/enhanced intermediate PET image for the next iteration. The details of network design and algorithm implementation could be found in our previous work [16].

### Network training and testing

One hundred patient data were collected for network training and testing. The injected dose of ^18^F-Fluorodeoxyglucose (FDG) was 3.7~4.4 MBq/kg, while the acquisition time was fixed at 900 s for TB-PET imaging with uEXPLORER. Benefits from its ultra-high sensitivity, uEXPLORER has demonstrated its ability to provide clinically acceptable images for a scan as short as 60 s and good images for a scan of more than 180 s. So a 900-s scan is long enough to generate excellent images with very low image noise and high image contrast. For CNN-DE, PET images with 10% uniformly down-sampled counts were used as training input, and PET images with full counts were used as training targets. For CNN-EH, PET images with insufficient iterations were used as training input, and PET images with sufficient iterations were used as training targets. The training image size was 249x249x671 with a voxel size of 2.4x2.4x2.68 mm^3^. The reconstruction algorithm was OP-OSEM with time-of-flight (TOF) and resolution modeling. All necessary corrections like scatter, normalization, dead time, attenuation, random, decay corrections were applied. Totally 53680 image pairs from 80 patients were used to construct the training dataset. The training data were augmented via flipping and rotating, and applied with z-score normalization before they were fed into the network.

The network parameters were initialized with Kaiming initialization. The loss function was the sum of the L1 losses of all three branches. The training of the network took the loss function as the objective function and the backpropagation algorithm was used to update the parameters based on the adaptive moment estimation optimization algorithm and cyclical learning rate. The minimum and maximum values for the cyclical learning rate were 1e-5 and 1e-4, respectively. The training settings were the same for both CNN-DE and CNN-EH. All the training was conducted using Pytorch 1.5.0 on a computer cluster of 4x NVIDIA Quadro RTX 6000 GPU. The CUDA library was 10.2, and cuDNN was 7. 6. The trained networks were tested with 13420 image pairs from the other 20 patients to ensure that they could be used in this study.


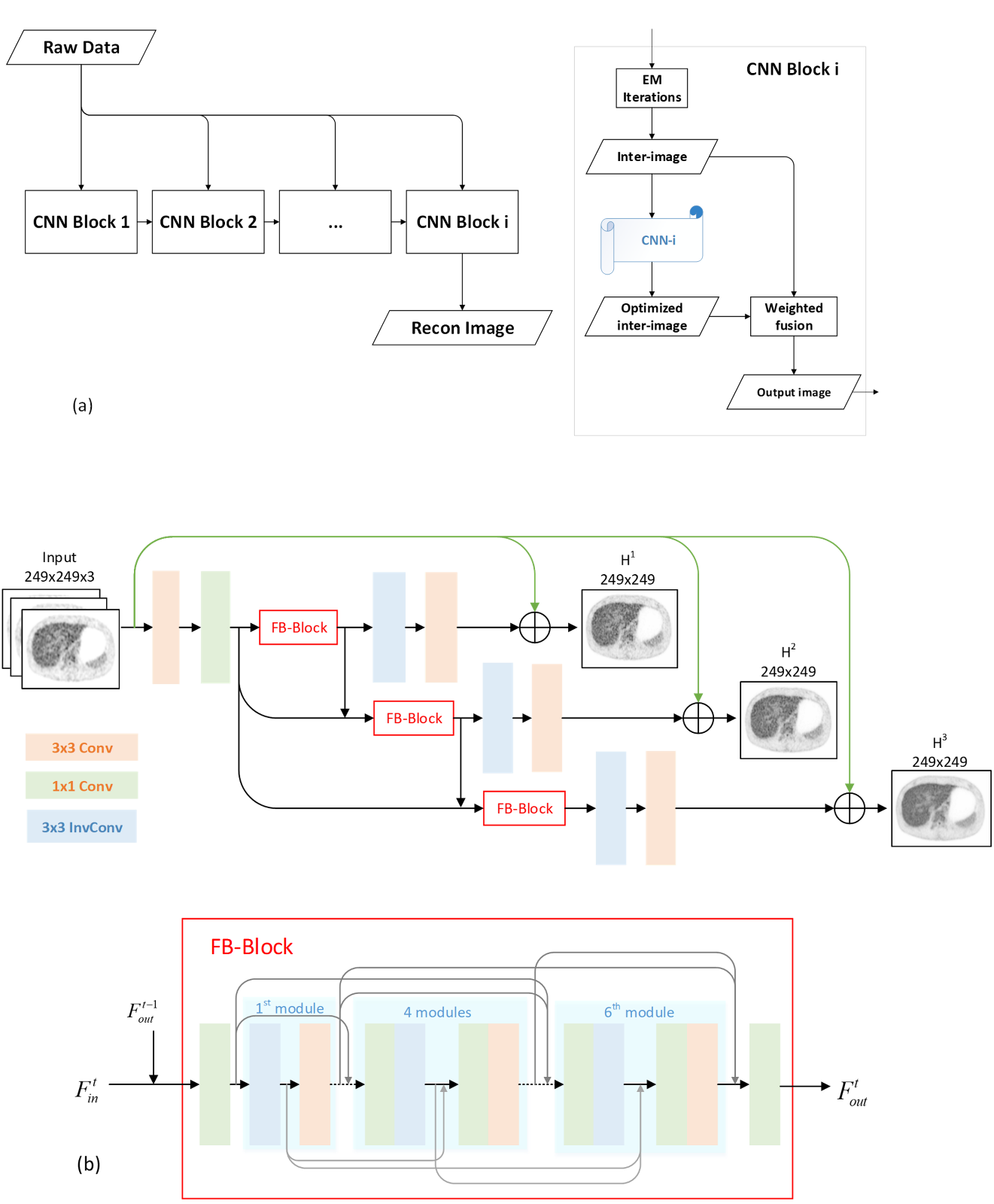


Fig. S1 (a) A general scheme of the DPR algorithm. The reconstruction workflow consists of multiple CNN blocks. Each block receives the output image of the previous block as the initial image for the EM iterations and passes the output image to the next CNN block. (b) The network architecture of the FB-Net. In this design, the network has three branches. H1, H2 and H3 are the outputs of these three branches respectively. Green lines indicate residual connections between the input and the output of each branch. Gray lines indicate dense connections between different layers in the FB-Block.
